# Supplementary material for: Complex‐centric proteome profiling by SEC‐SWATH‐MS
Source: Mol Syst Biol. 2019 Jan 14;15(1):e8438. doi: 10.15252/msb.20188438 (PMC6346213; doi:10.15252/msb.20188438)
Supplement: Supplementary file 7 — Dataset EV6 [file MSB-15-e8438-s007.zip › feature_plots_bioplex/P40429.pdf]

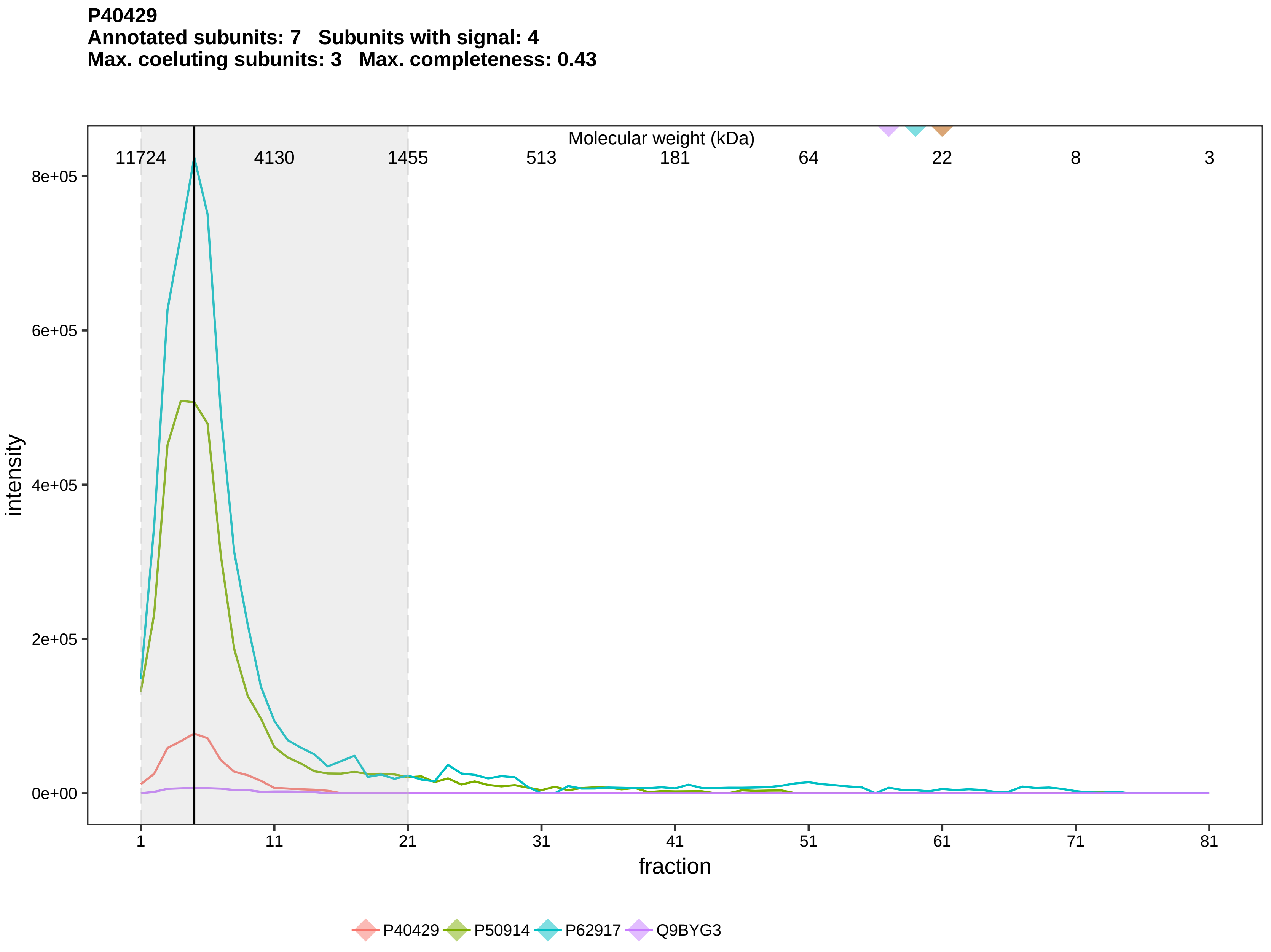
P40429
Annotated subunits: 7 Subunits with signal: 4
Max. coeluting subunits: 3 Max. completeness: 0.43
Molecular weight (kDa)
11724
4130
1455
513
181
64
22
8
3
intensity
8e+05
6e+05
4e+05
2e+05
0e+00
fraction
1
11
21
31
41
51
61
71
81
P40429
P50914
P62917
Q9BYG3
